# Supplementary material for: Development of a computer-aided design software for the quantitative evaluation of aesthetic damage
Source: PLoS One. 2019 Dec 18;14(12):e0226322. doi: 10.1371/journal.pone.0226322 (PMC6919621; doi:10.1371/journal.pone.0226322)
Supplement: S1 Code site — 1. (ZIP) [file pone.0226322.s001.zip › [EESC_jr][Projeto]Relatorio_07_21/reports/templates_zip/gentelella-master/vendors/jQuery-Smart-Wizard/smartwizard2-multiple.htm]

Smart Wizard 2 - Multiple Wizard Example - a javascript jQuery wizard control plugin

# Smart Wizard 3

## a javascript jQuery wizard control plugin

Download
Documentation
Github

### Examples:

Basic Example
Vertical Style
Multiple Wizards
Ajax Contents
Step Validation

## Example: Multiple Wizards

### Wizard 1

- 1

  Step 1  
  Step 1 description
- 2

  Step 2  
  Step 2 description
- 3

  Step 3  
  Step 3 description
- 4

  Step 4  
  Step 4 description

## Step 1 Content

- List 1
- List 2

Lorem ipsum dolor sit amet, consectetur adipisicing elit,
sed do eiusmod tempor incididunt ut labore et dolore magna aliqua. Ut enim ad minim veniam,
quis nostrud exercitation ullamco laboris nisi ut aliquip ex ea commodo consequat.
Duis aute irure dolor in reprehenderit in voluptate velit esse cillum dolore eu fugiat nulla pariatur.
Excepteur sint occaecat cupidatat non proident, sunt in culpa qui officia deserunt mollit anim id est laborum.

Lorem ipsum dolor sit amet, consectetur adipisicing elit,
sed do eiusmod tempor incididunt ut labore et dolore magna aliqua. Ut enim ad minim veniam,
quis nostrud exercitation ullamco laboris nisi ut aliquip ex ea commodo consequat.
Duis aute irure dolor in reprehenderit in voluptate velit esse cillum dolore eu fugiat nulla pariatur.
Excepteur sint occaecat cupidatat non proident, sunt in culpa qui officia deserunt mollit anim id est laborum.

Lorem ipsum dolor sit amet, consectetur adipisicing elit,
sed do eiusmod tempor incididunt ut labore et dolore magna aliqua. Ut enim ad minim veniam,
quis nostrud exercitation ullamco laboris nisi ut aliquip ex ea commodo consequat.
Duis aute irure dolor in reprehenderit in voluptate velit esse cillum dolore eu fugiat nulla pariatur.
Excepteur sint occaecat cupidatat non proident, sunt in culpa qui officia deserunt mollit anim id est laborum.

## Step 2 Content

Lorem ipsum dolor sit amet, consectetur adipisicing elit,
sed do eiusmod tempor incididunt ut labore et dolore magna aliqua. Ut enim ad minim veniam,
quis nostrud exercitation ullamco laboris nisi ut aliquip ex ea commodo consequat.
Duis aute irure dolor in reprehenderit in voluptate velit esse cillum dolore eu fugiat nulla pariatur.
Excepteur sint occaecat cupidatat non proident, sunt in culpa qui officia deserunt mollit anim id est laborum.

Lorem ipsum dolor sit amet, consectetur adipisicing elit,
sed do eiusmod tempor incididunt ut labore et dolore magna aliqua. Ut enim ad minim veniam,
quis nostrud exercitation ullamco laboris nisi ut aliquip ex ea commodo consequat.
Duis aute irure dolor in reprehenderit in voluptate velit esse cillum dolore eu fugiat nulla pariatur.
Excepteur sint occaecat cupidatat non proident, sunt in culpa qui officia deserunt mollit anim id est laborum.

Lorem ipsum dolor sit amet, consectetur adipisicing elit,
sed do eiusmod tempor incididunt ut labore et dolore magna aliqua. Ut enim ad minim veniam,
quis nostrud exercitation ullamco laboris nisi ut aliquip ex ea commodo consequat.
Duis aute irure dolor in reprehenderit in voluptate velit esse cillum dolore eu fugiat nulla pariatur.
Excepteur sint occaecat cupidatat non proident, sunt in culpa qui officia deserunt mollit anim id est laborum.

Lorem ipsum dolor sit amet, consectetur adipisicing elit,
sed do eiusmod tempor incididunt ut labore et dolore magna aliqua. Ut enim ad minim veniam,
quis nostrud exercitation ullamco laboris nisi ut aliquip ex ea commodo consequat.
Duis aute irure dolor in reprehenderit in voluptate velit esse cillum dolore eu fugiat nulla pariatur.
Excepteur sint occaecat cupidatat non proident, sunt in culpa qui officia deserunt mollit anim id est laborum.

## Step 3 Content

Lorem ipsum dolor sit amet, consectetur adipisicing elit,
sed do eiusmod tempor incididunt ut labore et dolore magna aliqua. Ut enim ad minim veniam,
quis nostrud exercitation ullamco laboris nisi ut aliquip ex ea commodo consequat.
Duis aute irure dolor in reprehenderit in voluptate velit esse cillum dolore eu fugiat nulla pariatur.
Excepteur sint occaecat cupidatat non proident, sunt in culpa qui officia deserunt mollit anim id est laborum.

Lorem ipsum dolor sit amet, consectetur adipisicing elit,
sed do eiusmod tempor incididunt ut labore et dolore magna aliqua. Ut enim ad minim veniam,
quis nostrud exercitation ullamco laboris nisi ut aliquip ex ea commodo consequat.
Duis aute irure dolor in reprehenderit in voluptate velit esse cillum dolore eu fugiat nulla pariatur.
Excepteur sint occaecat cupidatat non proident, sunt in culpa qui officia deserunt mollit anim id est laborum.

## Step 4 Content

Lorem ipsum dolor sit amet, consectetur adipisicing elit,
sed do eiusmod tempor incididunt ut labore et dolore magna aliqua. Ut enim ad minim veniam,
quis nostrud exercitation ullamco laboris nisi ut aliquip ex ea commodo consequat.
Duis aute irure dolor in reprehenderit in voluptate velit esse cillum dolore eu fugiat nulla pariatur.
Excepteur sint occaecat cupidatat non proident, sunt in culpa qui officia deserunt mollit anim id est laborum.

Lorem ipsum dolor sit amet, consectetur adipisicing elit,
sed do eiusmod tempor incididunt ut labore et dolore magna aliqua. Ut enim ad minim veniam,
quis nostrud exercitation ullamco laboris nisi ut aliquip ex ea commodo consequat.
Duis aute irure dolor in reprehenderit in voluptate velit esse cillum dolore eu fugiat nulla pariatur.
Excepteur sint occaecat cupidatat non proident, sunt in culpa qui officia deserunt mollit anim id est laborum.

Lorem ipsum dolor sit amet, consectetur adipisicing elit,
sed do eiusmod tempor incididunt ut labore et dolore magna aliqua. Ut enim ad minim veniam,
quis nostrud exercitation ullamco laboris nisi ut aliquip ex ea commodo consequat.
Duis aute irure dolor in reprehenderit in voluptate velit esse cillum dolore eu fugiat nulla pariatur.
Excepteur sint occaecat cupidatat non proident, sunt in culpa qui officia deserunt mollit anim id est laborum.

  

### Wizard 2

- 1

  Step 1  
  Step 1 description
- 2

  Step 2  
  Step 2 description
- 3

  Step 3  
  Step 3 description
- 4

  Step 4  
  Step 4 description

## Step 1 Content

- List 1
- List 2

Lorem ipsum dolor sit amet, consectetur adipisicing elit,
sed do eiusmod tempor incididunt ut labore et dolore magna aliqua. Ut enim ad minim veniam,
quis nostrud exercitation ullamco laboris nisi ut aliquip ex ea commodo consequat.
Duis aute irure dolor in reprehenderit in voluptate velit esse cillum dolore eu fugiat nulla pariatur.
Excepteur sint occaecat cupidatat non proident, sunt in culpa qui officia deserunt mollit anim id est laborum.

Lorem ipsum dolor sit amet, consectetur adipisicing elit,
sed do eiusmod tempor incididunt ut labore et dolore magna aliqua. Ut enim ad minim veniam,
quis nostrud exercitation ullamco laboris nisi ut aliquip ex ea commodo consequat.
Duis aute irure dolor in reprehenderit in voluptate velit esse cillum dolore eu fugiat nulla pariatur.
Excepteur sint occaecat cupidatat non proident, sunt in culpa qui officia deserunt mollit anim id est laborum.

Lorem ipsum dolor sit amet, consectetur adipisicing elit,
sed do eiusmod tempor incididunt ut labore et dolore magna aliqua. Ut enim ad minim veniam,
quis nostrud exercitation ullamco laboris nisi ut aliquip ex ea commodo consequat.
Duis aute irure dolor in reprehenderit in voluptate velit esse cillum dolore eu fugiat nulla pariatur.
Excepteur sint occaecat cupidatat non proident, sunt in culpa qui officia deserunt mollit anim id est laborum.

## Step 2 Content

Lorem ipsum dolor sit amet, consectetur adipisicing elit,
sed do eiusmod tempor incididunt ut labore et dolore magna aliqua. Ut enim ad minim veniam,
quis nostrud exercitation ullamco laboris nisi ut aliquip ex ea commodo consequat.
Duis aute irure dolor in reprehenderit in voluptate velit esse cillum dolore eu fugiat nulla pariatur.
Excepteur sint occaecat cupidatat non proident, sunt in culpa qui officia deserunt mollit anim id est laborum.

Lorem ipsum dolor sit amet, consectetur adipisicing elit,
sed do eiusmod tempor incididunt ut labore et dolore magna aliqua. Ut enim ad minim veniam,
quis nostrud exercitation ullamco laboris nisi ut aliquip ex ea commodo consequat.
Duis aute irure dolor in reprehenderit in voluptate velit esse cillum dolore eu fugiat nulla pariatur.
Excepteur sint occaecat cupidatat non proident, sunt in culpa qui officia deserunt mollit anim id est laborum.

Lorem ipsum dolor sit amet, consectetur adipisicing elit,
sed do eiusmod tempor incididunt ut labore et dolore magna aliqua. Ut enim ad minim veniam,
quis nostrud exercitation ullamco laboris nisi ut aliquip ex ea commodo consequat.
Duis aute irure dolor in reprehenderit in voluptate velit esse cillum dolore eu fugiat nulla pariatur.
Excepteur sint occaecat cupidatat non proident, sunt in culpa qui officia deserunt mollit anim id est laborum.

Lorem ipsum dolor sit amet, consectetur adipisicing elit,
sed do eiusmod tempor incididunt ut labore et dolore magna aliqua. Ut enim ad minim veniam,
quis nostrud exercitation ullamco laboris nisi ut aliquip ex ea commodo consequat.
Duis aute irure dolor in reprehenderit in voluptate velit esse cillum dolore eu fugiat nulla pariatur.
Excepteur sint occaecat cupidatat non proident, sunt in culpa qui officia deserunt mollit anim id est laborum.

## Step 3 Content

Lorem ipsum dolor sit amet, consectetur adipisicing elit,
sed do eiusmod tempor incididunt ut labore et dolore magna aliqua. Ut enim ad minim veniam,
quis nostrud exercitation ullamco laboris nisi ut aliquip ex ea commodo consequat.
Duis aute irure dolor in reprehenderit in voluptate velit esse cillum dolore eu fugiat nulla pariatur.
Excepteur sint occaecat cupidatat non proident, sunt in culpa qui officia deserunt mollit anim id est laborum.

Lorem ipsum dolor sit amet, consectetur adipisicing elit,
sed do eiusmod tempor incididunt ut labore et dolore magna aliqua. Ut enim ad minim veniam,
quis nostrud exercitation ullamco laboris nisi ut aliquip ex ea commodo consequat.
Duis aute irure dolor in reprehenderit in voluptate velit esse cillum dolore eu fugiat nulla pariatur.
Excepteur sint occaecat cupidatat non proident, sunt in culpa qui officia deserunt mollit anim id est laborum.

## Step 4 Content

Lorem ipsum dolor sit amet, consectetur adipisicing elit,
sed do eiusmod tempor incididunt ut labore et dolore magna aliqua. Ut enim ad minim veniam,
quis nostrud exercitation ullamco laboris nisi ut aliquip ex ea commodo consequat.
Duis aute irure dolor in reprehenderit in voluptate velit esse cillum dolore eu fugiat nulla pariatur.
Excepteur sint occaecat cupidatat non proident, sunt in culpa qui officia deserunt mollit anim id est laborum.

Lorem ipsum dolor sit amet, consectetur adipisicing elit,
sed do eiusmod tempor incididunt ut labore et dolore magna aliqua. Ut enim ad minim veniam,
quis nostrud exercitation ullamco laboris nisi ut aliquip ex ea commodo consequat.
Duis aute irure dolor in reprehenderit in voluptate velit esse cillum dolore eu fugiat nulla pariatur.
Excepteur sint occaecat cupidatat non proident, sunt in culpa qui officia deserunt mollit anim id est laborum.

Lorem ipsum dolor sit amet, consectetur adipisicing elit,
sed do eiusmod tempor incididunt ut labore et dolore magna aliqua. Ut enim ad minim veniam,
quis nostrud exercitation ullamco laboris nisi ut aliquip ex ea commodo consequat.
Duis aute irure dolor in reprehenderit in voluptate velit esse cillum dolore eu fugiat nulla pariatur.
Excepteur sint occaecat cupidatat non proident, sunt in culpa qui officia deserunt mollit anim id est laborum.
